# The Characteristic and Short-Term Prognosis of Tinnitus Associated with Sudden Sensorineural Hearing Loss

**DOI:** 10.1155/2018/6059697

**Published:** 2018-05-13

**Authors:** Xiaoqiong Ding, Xiaoli Zhang, Zhichun Huang, Xu Feng

**Affiliations:** ^1^Department of Otolaryngology, Head and Neck Surgery, Zhongda Hospital, Southeast University, No. 87 Dingjiaqiao Road, Nanjing 210009, China; ^2^Department of Otolaryngology, Head and Neck Surgery, Nanjing Drum Tower Hospital, No. 321 Zhongshan Road, Nanjing 210008, China

## Abstract

Tinnitus is believed to result from the maladaptive plasticity of the auditory nervous system; reports regarding its severity and prognosis are conflicting. We evaluated the characteristic and short-term prognosis of tinnitus associated with sudden sensorineural hearing loss (SSNHL). A total of 230 cases were enrolled. The severity and 1-month prognosis of tinnitus (according to the Tinnitus Handicap Inventory (THI)) were assessed in terms of the patients' sex, age, level of hearing loss, type of audiogram results, and so on. According to our statistical analysis, the degree of handicap due to tinnitus was not related to sex, age, or level of hearing loss; the Tinnitus Handicap Inventory indicated that the low-frequency-audiogram group had a low tinnitus handicap (*F* = 7.516, *P* = 0.000). Furthermore, we found that the prognosis of tinnitus was not related to the type of audiogram or level of hearing loss. Recovery from a severe level of hearing loss was, however, found to be associated with a poor tinnitus prognosis (*F* = 5.203, *P* = 0.006). In summary, our study indicates that the association between tinnitus and SSNHL is extremely high. Tinnitus can be ameliorated by the successful treatment of hearing loss. The study was registered in the Chinese Clinical Trial Registry (ChiCTR1800014797).

## 1. Introduction

Tinnitus, one of the most frequent sensorineural disorders, involves the perception of a fake sound in the absence of a corresponding sound stimulus; it is often considered as the result of maladaptive plasticity in the auditory system [1, 2]. It is reported that 5% to 10% of the population suffer from tinnitus and that it consequently has a negative impact in their life [3, 4]. On the other hand, sudden sensorineural hearing loss (SSNHL) is also a challenging clinical problem. It is reported that the incidence of SSNHL is as high as 5 to 20 per 100,000 people [5]. However, another research suggests a greater incidence of 160 per 100,000 people [6]. The association between tinnitus and SSNHL is extremely high (66% to 93%) [7, 8]. It has not yet been determined whether tinnitus is triggered only by hearing loss or whether the severity of tinnitus is affected by the level of hearing loss. It is also not clear whether the prognosis of hearing loss is associated with the prognosis of tinnitus. Some reports suggest that the prognosis for recovery from tinnitus does not conform with the recovery of hearing in patients with SSNHL. Others have reported that such a recovery is in fact relevant. The presence of tinnitus often has a highly negative impact on patients' lives, sometimes being considered even worse than the discomfort of hearing loss. Neural plasticity has played an important role in recovery from both the hearing loss and tinnitus. Acute tinnitus has been reported to have a high rate of spontaneous recovery; on the other hand, in some cases acute tinnitus has developed into chronic tinnitus, with an associated severely negative impact on patients' lives.

The purpose of the present research was to estimate the characteristics and prognosis of tinnitus associated with SSNHL and discuss the role of neural plasticity in tinnitus.

## 2. Materials and Methods

### 2.1. Ethics Statement

This research was approved by the Zhongda Hospital Southeast University Research Ethics Committee (Nanjing, China). Fully informed written consent for publication of clinical data was taken from each patient or from the guardians of the patients who were below 18 years of age.

### 2.2. Inclusion and Exclusion Criteria

A retrospective study was applied in patients suffering from tinnitus triggered by unilateral SSNHL who were treated at our hospital between January 2015 and July 2017; those with newly developed tinnitus were enrolled in our study. SSNHL was defined as more than 30 dB HL threshold shift in three contiguous frequencies or more in 72 hours. The cause of hearing loss was unknown; enrolled patients suffering from unilateral SSNHL were treated within 14 days of onset. The hearing loss and tinnitus were assessed before treatment and in the following 30 days.

### 2.3. Treatment

All patients received hyperbaric oxygen therapy, steroid, lidocaine, and ginaton (extract of *Ginkgo biloba* leaves injection, Dr. Willmar Schwabe Pharmaceuticals) administration. They were treated with hyperbaric oxygen therapy for 60 minutes once daily for 10 days, intravenous hydrocortisone sodium succinate (400 mg/day on days 1–3, 200 mg/day on days 4–6, and 100 mg/day on days 7–10), ginaton (87.5 mg/day for 10 days), and lidocaine (100 mg/day for 10 days). Patients with diabetes or hypertension were treated by intratympanic injection of prednol (20 mg/day for 10 days) instead of intravenous hydrocortisone.

### 2.4. Tinnitus Assessment

The evaluation of the impact of tinnitus on these patients' lives is very important and can be estimated through several questionnaires, the Tinnitus Handicap Inventory (THI) being one of the validated questionnaires. The THI questionnaire consists of three subscales: emotional (9 items), functional (11 items), and catastrophic (5 items). The yes answer to an item gets 4 scores or sometimes 2 scores, and a no gets a zero score. Scores of the total scale range from 0 to 100, with higher scores representing a greater perceived handicap [9, 10]. The severity of tinnitus as measured by the total score is classified as negligible (0–16), mild (18–36), moderate (38–56), severe (58–76), or catastrophic (78–100) [11]. The THI questionnaire was used to estimate the severity of tinnitus before treatment and in the 30-day period after onset. The prognosis of tinnitus was considered as effective if the THI score was improved more than 10 and noneffective if the THI score was improved no more than 10.

### 2.5. Classification of Level of Hearing Loss

Pure-tone threshold was measured in the affected and nonaffected ears. Pure-tone average thresholds at 0.5, 1, 2, and 4 kHz were assessed as mean pure-tone threshold. When the hearing thresholds of deep losses were not detected, the threshold was considered as the maximum audiometric intensity. Hearing loss was classified into 5 degrees according to the mean pure-tone threshold: mild (26–40 dB HL), moderate (41–55 dB HL), moderately severe (56–70 dB HL), severe (71–90 dB HL), and profound (>90 dB HL). Pure-tone threshold was measured before treatment and during the 30-day period after onset.

### 2.6. Classification of Audiogram

The audiogram was classified into 4 types by the method reported in[12]. The audiograms were categorized into (1) type A, low-frequency type, hearing loss in low-tone frequencies (250, 500, and 1000 Hz) was at least 15 dB HL more than the other frequencies; (2) type B, flat type, hearing loss is no more than 15 dB HL between low-tone frequencies (250, 500, and 1000 Hz) and high frequencies (above 2000 Hz); (3) type C, high-frequency type, hearing loss in the high frequencies (above 2000 Hz) at least 15 dB HL more than other frequencies; and (4) type D, total deafness, hearing loss of 81 dB HL or more in all frequencies.

### 2.7. Hearing Recovery Criteria

The following criteria [12] were used to assess hearing recovery: (1) cure: affected frequencies return to within 10 dB of the unaffected ear or normal; (2) obviously effective recovery: affected frequencies are at threshold recovery greater than 30 dB HL at mean; (3) effective recovery: affected frequencies are at threshold recovery greater than 15 dB HL at mean; and (4) no effective: affected frequencies are at threshold recovery no more than 15 dB HL at mean. For purposes of statistical analysis of the data from this study, patients with obviously effective recovery and effective recovery were grouped in the same group, called the “effective group.”

### 2.8. Statistical Methods

Statistical analyses were performed with SPSS software (IBM Corp., version 22). Tests and graphs were based on analysis sets that included all patients. The severity of tinnitus (THI) in terms of patients' sex, age, audiogram type, and hearing loss level was studied by Ridit analysis. The prognosis of tinnitus in terms of audiogram type, level of hearing loss, prognosis of hearing loss, and the severity of tinnitus (THI) was also studied by the same method.

## 3. Results

Of a total of 283 unilateral SSNHL patients enrolled within 14 days of the onset of acute hearing loss, 252 (89.0%) reported the new development of tinnitus. Of these 252, a total of 22 patients were lost to follow-up. The mean age of the 230 assessed patients was 43.5 years, with a median of 45 years, standard deviation of 15.1; minimum of 15 years and maximum of 70 years. Of those assessed, 136 patients were men (59.1%) and 94 were women (40.9%). There were 22 cases of negligible tinnitus (9.57%), 96 mild cases (41.74%), 86 moderate cases (37.39%), 22 severe cases (9.57%), and 4 catastrophic cases (1.74%).

Sex, age, initial hearing loss level, and audiogram type were reported to be associated with the severity of tinnitus. The degrees of difference in THI in terms of sex and age were compared by Ridit analysis (Table 1). The patients were divided into 5 age groups: 15 to 30 (53 cases, 23.04%), 31 to 40 (38 cases, 16.52%), 41 to 50 (58 cases, 25.22%), 51 to 60 (47 cases, 20.43%), and 61 to 70 (34 cases, 14.78%). Statistically, there was no significant difference between males and females (*P* = 0.600). There was also no significant difference between age groups (*P* = 0.598).

The THI and audiogram scores at different levels of hearing loss were compared by Ridit analysis (Table 2). In terms of the hearing loss, there were 71 mild and moderate cases (30.87%), 34 moderately severe cases (14.78%), 59 severe cases (25.65%), and 66 profound cases (28.70%). There was no significant difference among different hearing loss levels in terms of the THI score (*F* = 0.704, *P* = 0.550). However, there was a significant difference among different audiograms (*F* = 7.516, *P* = 0.000). The low-frequency-audiogram group had a lower score on the THI compared with others.

Tinnitus prognosis was assessed by the THI score. Of the enrolled patients, there were 182 effective cases (79.13%) and 48 noneffective cases (20.87%). The tinnitus prognosis was compared in terms of audiogram result, hearing loss level, hearing prognosis, and THI score. There was no significant difference in tinnitus prognosis among the different audiogram types (*F* = 1.640, *P* = 0.181) (Table 3). However, there was a significant difference among the different hearing prognoses (*F* = 5.203, *P* = 0.006). Patients of the no effective group had a poor tinnitus prognosis compared to the cure group and effective group (Table 4).

The initial level of hearing loss has frequently been considered a prognostic factor for tinnitus. We used one-way orderly Ridit statistical analysis to determine whether there was a correlation between the initial level of hearing loss and the prognosis of tinnitus. There was no significant difference among different hearing loss levels (*F* = 0.170, *P* = 0.917) (Table 5). However, there was a significant difference between the different degrees of THI (*F* = 10.623, *P* = 0.000). The initial degree of THI was also compared with the final degree of THI. Patients with moderate and severe degrees of THI had better tinnitus prognosis than others (Table 6).

## 4. Discussion

The generation and maintenance of tinnitus are challenging topics of neural research [4, 13]. The mechanism of tinnitus has been studied, and great progress has been made in recent decades. And till now, it has been found that tinnitus is produced in the brain and not in the ear. A study has recently focused on studying how tinnitus might be generated by plasticity and aberrant processing in the peripheral and central auditory system. Much research has been devoted to elucidating the relationship between tinnitus and hearing loss. Acute tinnitus is triggered by cochlear impairment. The reduction signal transduction from the impaired cochlea is considered to reduce lateral inhibition in the brainstem auditory pathway, such as the dorsal cochlear nucleus or inferior colliculus, finally resulting in the high spontaneous activity of auditory neurons around the impaired frequencies [4, 14].

In most patients, tinnitus is associated with hearing loss [8, 15, 16]. The association between tinnitus and SSNHL is extremely high (66 to 93%) [7, 8]. The relationship between tinnitus and hearing loss has also been proved in animal studies [17–20]. Tinnitus in SSNHL was an extremely frequent symptom as reported in the literature and also in our study: 89.0% (252 of 283). Its impact on patients' quality of life was also highly noticeable. However, hearing loss is not always associated to tinnitus. As reported, 7% to 34% patients suffering from SSNHL did not complain of tinnitus [7, 8]. In our study, 11% (31 of 283) of the patients did not complain of tinnitus accompanying SSNHL. In scrutinizing the connection between tinnitus and hearing loss, many researchers' measurements have been based on combined techniques. A recent study showed that subjects with tinnitus as well as hearing loss had better outer hair cell (OHC) function than subjects without tinnitus [21]. When assessed at a higher probe level, the psychophysical tuning lines of the subjects with tinnitus were consistent with those of subjects with normal hearing, implying that OHC damage in subjects with tinnitus might not be as severe as previously thought. Inner hair cell (IHC) damage or auditory nerve fiber dysfunction may play a more important role in the emergence of tinnitus. Auditory brainstem responses of tinnitus subjects with normal audiograms show a significantly decreased amplitude of the wave I potential but normal amplitudes of the wave V. These phenomena have been found in subjects with tinnitus and normal pure-tone hearing threshold [22, 23].

The severity of tinnitus can be influenced by cochlea damage-induced neuronal plasticity throughout the auditory nervous system from the hair cells to the auditory cortex [24] and also by the patient's psychological condition and educational background [25, 26]. Studies have demonstrated that the severity of tinnitus (THI) is not related to sex, age, degree of hearing loss, or audiogram type [27]. We found that the degree of THI was not related to sex or degree of hearing loss, but we did not identify the patients' psychological condition or educational background. We found that the degree of THI was related to the audiogram type and that the low-frequency-audiogram group had a low degree of THI compared with others. Patients with SSNHL and tinnitus ordinarily match the tinnitus pitch to the impaired frequencies or to the audiogram edge [4, 28]. Tinnitus pitch was focused in low frequencies in low-frequency-audiogram SSNHL; this type of tinnitus could easily be covered by environmental noise. Therefore, it may be that the tinnitus of patients with low-frequency-audiogram SSNHL tends to be of lower severity.

It has been found that tinnitus can be treated effectively by treating the concomitant conductive hearing loss [29–31]. Tinnitus was cured when conductive hearing loss was relieved, in more than half of the subjects, and most of the remaining subjects experienced improvement [29, 30]. Tinnitus was improved in more than 80% of the patients who underwent tympanoplasty [31]. Tinnitus can be ameliorated by the use of an artificial cochlear implant and hearing aid [32, 33]. It can also be reduced by treating sensorineural hearing loss [4, 8, 16, 34–36]. Nogueira-Neto et al. have demonstrated that the smaller the THI gain, the greater the degree of hearing recovery [8]. Another study reported that the degree of tinnitus improvement was consistent with SSNHL improvement [16]. Cure of hearing loss and tinnitus were both about three times more frequent in patients with mild to moderate hearing loss than in those with severe to profound categories. It has been found that the pure-tone threshold and speech discrimination score after SSNHL treatment were significantly improved in the patients with significant tinnitus as compared with those whose tinnitus was less severe [35]. Our research has demonstrated that an improvement in tinnitus is associated with the amelioration or cure of SSNHL. There was a significant correlation between tinnitus improvement and hearing recovery after treatment for SSNHL, whereas there was no relation between the degree of hearing loss and the prognosis of tinnitus. Moreover, there is no correlation between initial audiogram types and tinnitus prognosis. This implies that hearing recovery may be a prognostic factor of accompanying tinnitus in SSNHL; that is, the accompanying tinnitus can be improved by the successful treatment of SSNHL.

Our study found that hearing recovery is consistent with a decrease in tinnitus, although the initial level of hearing loss or audiogram type is not a prognostic factor for tinnitus. A study has also shown earlier effects on hearing recovery than on complete tinnitus remission in patients with severe-profound hearing loss [37]. Hearing loss and tinnitus are caused by damage to or dysfunction of the auditory system [38, 39], and there seems to be a difference between the capacity for recovery of afferent input for hearing and for resolution of the perception of tinnitus. The tinnitus recovery mechanism is different from the mechanism for hearing recovery. Many useful methods have been studied to protect the cochlea from damage [40, 41], and several effective methods have been researched to protect the cochlea in vitro [42–46]. However, there were limited methods to treat tinnitus [47]. Generally, sensorineural hearing loss tends to be stable within 3 months of onset, whereas there can be a spontaneous decrease in tinnitus within 5 years of onset. Tinnitus is the result of maladaptive plasticity within the auditory system [1, 14, 18]; the reversal of such changes in plasticity takes longer than the intrinsic cochlear repair mechanisms and may actually depend to some extent on them.

The results of the present study suggest that the association between tinnitus and SSNHL is extremely high. Tinnitus associated with SSNHL has a negative impact on patients' quality of life. The tinnitus was less severe in the low-frequency-audiogram group compared with other types. Tinnitus can be ameliorated by the treatment of SSNHL. Maladaptive plasticity plays an important role in the mechanism of tinnitus. However, the present research reports the short-term prognosis of tinnitus as associated with SSNHL; the longer-term prognosis requires further analysis to assess the neural plasticity of the cochlea and the brain.

## Figures and Tables

**Table 1:** The severity of tinnitus in terms of sex and age.

Classifications	Severity of tinnitus (THI)					
	No. of cases	Negligible	Mild	Moderate	Severe	Catastrophic
Sex						
Male	136	12	50	57	15	2
Female	94	10	46	29	7	2
Age						
15–30	53	10	18	17	8	0
31–40	38	5	15	13	3	2
41–50	58	2	27	22	7	0
51–60	47	2	18	24	2	1
61–70	34	3	18	10	2	1

THI, Tinnitus Handicap Inventory. There was no significant difference between males and females (*P* = 0.600) or between age groups (*P* = 0.598).

**Table 2:** Severity of tinnitus in patients with hearing loss.

Classifications of hearing loss	Severity of tinnitus (THI)					
	No. of cases	Negligible	Mild	Moderate	Severe	Catastrophic
Hearing loss level						
Mild and moderate	71	9	29	26	7	0
Moderately severe	34	3	18	9	4	0
Severe	59	5	23	25	5	1
Profound	66	5	26	26	6	3
Audiogram type						
Type A, low frequency	28	7	18	2	1	0
Type B, flat	83	9	32	34	8	0
Type C, high frequency	65	3	24	30	7	1
Type D, total deafness	54	3	22	20	6	3

THI, Tinnitus Handicap Inventory. In terms of the degree of THI, there was no significant difference among the levels of hearing loss (*F* = 0.704, *P* = 0.550). However, there was a significant difference between different audiograms (*F* = 7.516, *P* = 0.000). The low-frequency-audiogram group had low THI scores compared with others (*P* = 0.000).

**Table 3:** Tinnitus prognosis and hearing loss curve.

Audiogram type	Tinnitus prognosis	
	Effective group	Noneffective group
Type A, low frequency	25	3
Type B, flat	68	15
Type C, high frequency	46	19
Type D, total deafness	43	11

There was no significant difference among different audiogram types (*F* = 1.640, *P* = 0.181).

**Table 4:** The prognosis of tinnitus as related to the prognosis of hearing loss.

Hearing prognosis	Tinnitus prognosis	
	Effective group	Noneffective group
Cure group	41	4
Effective group	68	13
No effective group	73	31

There was a significant difference among the different hearing prognoses (*F* = 5.203, *P* = 0.006). Patients of the no effective group had a poor tinnitus prognosis than the cure group (*P* = 0.004) and effective group (*P* = 0.021).

**Table 5:** Tinnitus prognosis and level of hearing loss.

Level of hearing loss	Tinnitus prognosis	
	Effective group	Noneffective group
Mild to moderate	57	14
Moderately severe	26	8
Severe	48	11
Profound	51	15

There was no significant difference among the different levels of hearing loss (*F* = 0.170, *P* = 0.917).

**Table 6:** Tinnitus prognosis and THI.

Tinnitus prognosis	Severity of tinnitus (THI)					
	Cases	Negligible	Mild	Moderate	Severe	Catastrophic
Effective group	182	11	65	81	22	3
No effective group	48	11	31	5	0	1

THI, Tinnitus Handicap Inventory. There was significant difference among different THI degrees (*F* = 10.623, *P* = 0.000). Mild cases had a better prognosis than negligible cases (*P* = 0.048). Moderate cases had a better tinnitus prognosis than negligible and mild cases (*P* = 0.000). Severe cases had a better prognosis than negligible and mild cases (*P* = 0.000).

## Data Availability

The data used to support the findings of this study are available from the corresponding author upon request.
